# A new method to measure ligament balancing in total knee arthroplasty: laxity measurements in 100 knees

**DOI:** 10.1007/s00402-012-1536-1

**Published:** 2012-05-13

**Authors:** Eirik Aunan, Thomas Kibsgård, John Clarke-Jenssen, Stephan M. Röhrl

**Affiliations:** 1 Department of Surgery, Innlandet Hospital Trust, Anders Sandvigs Gate 17, 2629 Lillehammer, Norway; 2 Department of Orthopedics, Oslo University Hospital, Postboks 4950, Oslo, Norway

**Keywords:** Total knee arthroplasty, Ligament balance, Soft tissue balance, Flexion–extension gap, Surgical technique, Equipment design

## Abstract

**Background:**

Ligament balancing is considered a prerequisite for good function and survival in total knee arthroplasty (TKA). However, there is no consensus on how to measure ligament balance intra-operatively and the degree of stability obtained after different balancing techniques is not clarified.

**Purpose:**

This study presents a new method to measure ligament balancing in TKA and reports on the results of a try-out of this method and its inter-observer reliability.

**Methods:**

After the implantation of the prosthesis, spatulas of different thickness were used to measure medial and lateral condylar lift-off in flexion and extension in 70 ligament-balanced knees and in 30 knees were ligament balancing was considered unnecessary. Inter-observer reliability for the new method was estimated and the degree of medial–lateral symmetry in extension and in flexion, and the equality of the extension gaps and flexion gaps were calculated.

**Results:**

The method was feasible in all operated knees, and found to be very reliable (intraclass correlation coefficient = 0.88). We found no statistically significant difference in condylar lift-off between the ligament-balanced and the non ligament-balanced group, however, there was a tendency to more outliers in flexion in the ligament-balanced group.

**Conclusions:**

Our method for measuring ligament balance is reliable and provides valuable information in assessing laxity intra-operatively. This method may be a useful tool in further research on the relationship between ligament balance, function and survival of TKA.

## Introduction

Symmetric ligament balance is considered a prerequisite for good function and endurance in total knee arthroplasty (TKA) [1–4]. Lack of medial–lateral symmetry in the flexion or extension gaps, or both, may lead to instability, poor function and wear. Inequality between the flexion gap and the extension gap may cause decreased range of motion or instability.

The immediate consequences of poor ligament balance differ depending on the implantation technique. If measured resection technique is used poor ligament balance can lead to asymmetric medial and lateral condylar lift-off. If the balanced gap technique is used, the ligament balance in flexion will influence on the rotation of the femoral component [5, 6].

Many surgical techniques for ligament balancing have been developed [7–15], and different devices designed to assist in ligament balancing have emerged. These include spacers [9], tensors [9, 16, 17], electronic instruments [18–22], and computers [23–27]. Despite the availability of these devices, defining optimal ligament tension during TKA is still mostly based on the surgeons “feel” and personal experience. Proper intra-operative laxity is typically judged subjectively, rather than measured [1, 28]. We believe one reason for this may be a lack of a simple method to measure ligament balance during surgery. There is also little objective information in the literature to what degree ligament balance can be achieved by different techniques for soft tissue release.

The primary goal of this study is to introduce a new, simple method to measure medial and lateral condylar lift-off in extension and in 90° of flexion intra-operatively during TKA. The inter-observer reliability of the new method is measured.

The second goal is to report on the results of the direct measurements, the degree of medial–lateral symmetry in extension and in flexion, and the equality of the extension gaps and flexion gaps in 70 ligament-balanced and 30 non ligament-balanced TKAs.

## Patients and methods

One-hundred knees in 90 patients, of which 56 were women, were operated consecutively. Patient demographics and Knee Society score (KSS) at baseline are shown in Table 1. Details of preoperative alignment and deformity are summarized in Table 2.

**Table 1:** Patient demographics and Knee Society Score (KSS) at baseline divided in groups with and without ligament balancing

Variable	Without ligament balancing (*n* = 30)	With ligament balancing (*n* = 70)	*p* value	Total
Gender (female)	17 (56.7 %)	39 (55.7 %)		100
Age^a^	71.0 (7.3) 53 to 83	69.2 (8.4) 42–81	0.30	69.7 (8.1) 42–83
BMI^a^	28.8 (3.5) 22 to 34	29.5 (4.0) 23–43	0.41	29.3 (3.9) 22–43
KSS knee score^a^	36.3 (20.4) −5 to 95	31,9 (14.3) 5–67	0.22	33.2 (16.4) −5–95
KSS function score^a^	64.8 (18.5) 30 to 100	64.9 (20.6) 30–100	0.98	64.9 (19.9) 30–100

^a^Data are presented as means, (SDs), and ranges

**Table 2:** Alignment and deformity at baseline divided in groups with and without ligament balancing

Alignment	Without ligament balancing		With ligament balancing		*p* value	Total
	*n*	Deformity^a^	*n*	Deformity^a^		
Varus knees	18	7.4 (5.2) 1–21	63	10.0 (4.5) 3–22	0.04	81
Valgus knees	9	5.9 (1.8) 3–9	6	5.0 (1.8) 2–7	0.37	15
Neutral knees	3	0	1	0	–	4
Total	30		70			100

^a^Deformity was measured in degrees and defined as the deviation from the ideal mechanical axis on HKA X-rays. Data are presented as means, (SDs), and ranges

All patients were consecutively recruited from another ongoing prospective, randomized and double-blind study (comparing patella resurfacing to no resurfacing). Inclusion criteria were patients <85 years scheduled for TKA because of osteoarthritis. Exclusion criteria were knees with severe deformity not suitable for standard cruciate-retaining prosthesis, rheumatoid arthritis, patellar thickness below 18 mm and severe medical disability limiting the ability to walk. The protocol was approved by the Regional Committee of Research Ethics, and before enrolment, all patients signed an informed-consent form. Operations were undertaken between October 2007 and November 2010 in a community hospital doing about 50 TKAs per year. To assure conformity in surgical technique, the first author (EA) was either operating or assisting in every operation.

### Surgical technique

All knees were operated through a standard midline incision and a medial parapatellar arthrotomy, using a cruciate-retaining prosthesis (NexGen, Zimmer, Warsaw, IN, USA). We used measured resection technique which involves resecting the amount of bone from the distal and posterior femur and the proximal tibia that will be replaced by the prosthetic components. The valgus angle of the femoral component was set at 5–8°, depending on the hip–knee–femoral shaft angle (HKFS) as measured on preoperative standing hip–knee–ankle (HKA) X-rays. Rotation of the femoral component was established by combining information from the anterior-posterior axis of the femur (Whiteside’s line), the transepicondylar line and the posterior condylar line. Osteophytes were resected. With an intramedullary guide in the femur and an extramedullary guide on the tibia, saw-blocks were fit into place. After the saw cuts were performed, posterior osteophytes were removed. With a trial prosthesis implanted, the ligament balance was evaluated. If asymmetric, the knee was balanced using the technique described by Whiteside, Saeki, Mihalko, Kanamiya et al. [12, 13, 29, 30]. The aims of the ligament balancing were medial and lateral condylar lift-off of 1–3 mm in both extension and 90° of flexion, and equal and rectangular flexion and extension gaps. When forced to choose, we went for a bigger gap laterally and/or in flexion. If anterior lift-off was observed in less than 100° of flexion, after ligament balancing was accomplished, the posterior cruciate ligament was released with a small tibial bone block. If there was a persistent mismatch between the extension and the flexion gap of more than 5 mm, additional bone cuts, according to the contingency table proposed by Mont and Delanois [31], were performed. All operations were performed in bloodless field with a tourniquet on the proximal part of the thigh.

### The new method to measure ligament balance

**Fig. 1:**
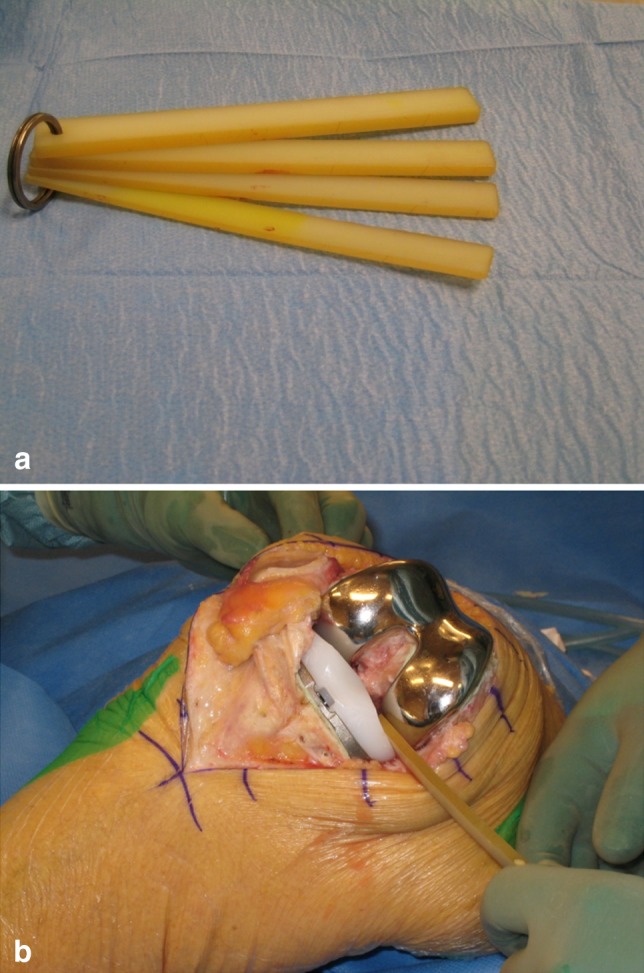
**a** The tool for measuring condylar lift-off consists of four spatulas made of polyethylene, from 2 to 5 mm thick. **b** With the knee in 90° of flexion medial condylar lift-off was defined as the distance in the frontal plane from the deepest point of the polyethylene tray to the most posterior point of the femoral condyle. The measurement was performed with the leg in a reversed crossed-leg position under passive varus-stress from the weight of the lower leg with the thickest spatula that could be introduced without force

After implantation of the prosthesis, we used a set of four polyethylene spatulas with thicknesses from 2 to 5 mm to measure the medial and lateral gaps (Fig. 1a). With the knee in extension, lift-off was defined as the distance in the frontal plane from the deepest point of the polyethylene tray to the most distal point of the femoral condyle. With the knee in 90° of flexion, the same measurements were done between the deepest point of the polyethylene tray to the most posterior point of the femoral condyle. With the knee in extension, the surgeon stressed the ligaments in valgus and varus until a firm endpoint was felt. Lift-off was measured by inserting the thickest spatula possible (Fig. 1b). If the thinnest spatula could not be inserted and there still was a visible gap, the gap was recorded as 1 mm, in the case of no visible gap, 0 mm was recorded. If the gap was more than 5 mm two spatulas were appositioned. In flexion, measurements were performed in the positions described by Tokuhara et al. [32]: lateral lift-off in 90° of flexion was measured in the unilateral cross-legged position under passive valgus stress by the weight of the lower leg. Medial lift-off in flexion was measured in a similar way with the leg in a reversed cross-leg position (Fig. 1b).

### Measurements

Medial and lateral lift-off was measured in extension and in 90° of flexion, and then, medial–lateral symmetry in extension and in flexion was calculated. The difference in size between the extension and the flexion gap was calculated by subtracting the mean values of medial and lateral lift-off in flexion from the mean values of the medial and lateral lift-off in extension. In all knees, the measurements were done with the patella everted.

### Inter-observer reliability

To evaluate the reliability of the method, an inter-rater analysis was performed in 96 consecutive measurements (24 knees). First the assisting surgeon measured the gaps while the operating surgeon stressed the knee ligaments. To assure blinding between the observers, the operating surgeon turned his head away from the field while the measurements were performed by the assisting surgeon. The results of the measurements were communicated to the circulating nurse by finger signs. Thereafter, the two surgeons changed roles. Four different assistants with very dissimilar experience in total knee surgery and the senior surgeon performed the measurements in this part of the study.

### Statistics

Data were stored and analyzed with use of Microsoft Access^®^ (Microsoft, Redmond, Washington, USA) and SPSS^®^ software (SPSS, Chicago, IL, USA). To determine inter-observer agreement between raters of condylar lift-off intraclass correlation statistics for single measures was performed. The distribution of data on condylar lift-off was analyzed with a Kolmogorov–Smirnov test. For the comparisons of lift-off between ligament-balanced and non ligament-balanced knees, we used the independent samples test for normally distributed data and the Mann–Whitney test for skewed data.

## Results

### Inter-observer reliability

Inter-observer agreement among raters was high with an intraclass correlation coefficient for single measures of 0.88 (95 % confidence interval 0.82–0.92). Absolute agreement was achieved in 60.4 % of measurements. In only one case, the difference between observers reached 2 mm.

### Ligament-balancing procedures

In 70 out of 100 knees, ligament balancing was undertaken, and in 30 knees, ligament surgery was deemed unnecessary. Among the ligament-balanced knees, 63 knees were deformed in varus, 6 were deformed in valgus and 1 knee was without preoperative deformity. The numbers of ligaments that were released in the varus- and valgus-deformed knees are presented in Table 3. Although the deep and superficial medial collateral ligaments are two anatomical structures, the current technique for ligament balancing regards the two layers as one functional unit with an anterior part that tightens in knee flexion and a posterior part that tightens in knee extension [12].

**Table 3:** Number of ligaments released in varus and valgus deformed knees

Ligament	Varus knees (*n *= 63)	Valgus knees (*n *= 6)
MCL		
Anterior part	49	
Posterior part	39	
Medial posterior capsule	10	
Semimembranosus	2	
Pes anserinus	–	–
PCL	27	3
LCL	1^a^	1
Popliteus tendon	4^a^	3
Posterolateral corner		1
Iliotibial tract		2
Lateral posterior capsule		2

*MCL* medial collateral ligament, *PCL* posterior cruciate ligament, *LCL* lateral collateral ligament

^a^Compensatory release in varus knees

Additional bone cuts were performed in ten cases; six re-cuts on the tibia, one recut on the distal femur and three cases of downsizing of the femur.

### Laxity measurements

There was no statistically significant difference between ligament-balanced knees and non ligament-balanced knees in medial and lateral condylar lift-off in extension and 90° of flexion for varus and valgus knees (Table 4).

**Table 4:** Medial and lateral lift-off in extension and 90° of flexion in knees with or without ligament balancing

Knee alignment	Position	Without ligament balancing	With ligament balancing	*p* value	Total
Varus knees		*n* = 18	*n* = 63		81
	Extension				
	Medial	1.6 (1.2–1.9) 1–3	1.9 (1.7–2.1) 1–4	0.17	
	Lateral	2.0 (1.6–2.4) 1–3	2.1 (1.9–2.3) 1–5	0.90	
	Flexion				
	Medial	2.7 (2.2–3.2) 0–4	3.4 (2.9–3.9) 1–9	0.30	
	Lateral	3.2 (2.6–3.9) 1–5	3.5 (3.1–3.9) 1–10	0.74	
Valgus knees		*n* = 9	*n* = 6		15
	Extension				
	Medial	2.0 (1.6–2.4) 1–3	2.7 (1.4–3.9) 1–4	0.25	
	Lateral	1.7 (1.1–2.2) 1–3	1.7 (0.2–3.1) 0–4	1.00	
	Flexion				
	Medial	2.4 (1.6–3.3) 1–4	3.7 (0.9–6.5) 1–8	0.33	
	Lateral	3.0 (1.7–4.3) 1–7	4.3 (2.9–5.8) 2–6	0.12	
Neutral knees		*n* = 3	*n* = 1		4
	Extension				
	Medial	2.3 (–) 1–3	1.0		
	Lateral	1.3 (–) 0–3	3.0		
	Flexion				
	Medial	3.3 (–) 2–4	2.0		
	Lateral	2.3 (–) 0–5	3.0		

With the knee in extension the surgeon stressed the collateral ligaments until a firm endpoint. Lift-off was defined as the distance in the frontal plane from the deepest point of the polyethylene tray to the most distal point of the femoral condyle. With the knee in 90° of flexion, the same measurements were done between the deepest point of the polyethylene tray and the most posterior point of the femoral condyle while the collateral ligaments were stressed by gravity (see text). Values are expressed in millimeters as means, (95 % CIs), and ranges

**Fig. 2:**
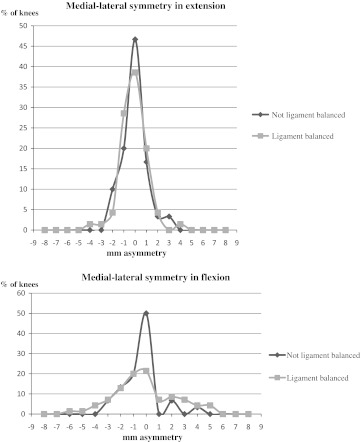
The degree of medial–lateral symmetry in lift-off that was achieved after implantation of the prosthesis, in knees where ligament balancing was not necessary (*n *= 30) and in knees that were ligament balanced according to the Whiteside method (*n* = 70). *Negative values* represent more lift-off laterally than medially. *Positive values* mean more lift-off medially than laterally

In extension, medial–lateral symmetry within 2 mm was obtained in 96 % of the knees undergoing ligament balancing and in 97 % of the knees not undergoing ligament balancing (Fig. 2). In flexion, medial–lateral symmetry within 2 mm was obtained in 70 % of the ligament-balanced knees and in 89 % of the knees without ligament balancing (Fig. 2).

**Fig. 3:**
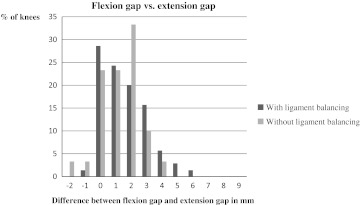
The relationship between the flexion gap and the extension gap. *Positive values* mean the flexion gap is larger than the extension gap. *Negative values* mean the extension gap is larger. *Zero* means the two gaps are of equal size

Flexion gaps were equal to extension gaps in 29 % of the ligament-balanced knees and in 23 % of the knees where no ligament surgery was performed (Fig. 3). In the knees with unequal gaps, 98 % of the ligament-balanced knees were tightest in extension and 91 % of the non ligament-balanced knees were tightest in extension (Fig. 3).

### Complications

Three intra-operative complications occurred. In one case, the popliteus tendon was cut, probably by the oscillating saw, while performing the posterior, lateral bone cut. In another case, the medial collateral ligament was damaged by the saw when performing the proximal tibial bone cut. The last was an inadvertent saw cut to the posterior cruciate ligament.

## Discussion

### The new method

Our method measures, intra-operatively directly in millimeters, medial and lateral condylar lift-off in extension and 90° of flexion. We consider the measuring procedure as easy to perform, and the measurements take no more than 1 or 2 min.

One might argue that this method will not give an accurate and reproducible tension to the ligaments during measurements of condylar lift-off. However, in every knee in this study, there was a firm endpoint when exposed to valgus and varus stress, and there is some evidence in the literature that clinician-applied stress to quantify the lift-off is quite reliable. LaPrade et al. [33] compared the lateral compartment gapping on stress radiographs before and after sequential lateral ligament sectioning in ten cadavers. Varus stress was applied either by a clinician or by a force-application device delivering a 12 Nm moment to the knee. They concluded that both standardized 12 Nm moments and clinician-applied varus stress radiographs provide objective and reproducible measures of lateral compartment gapping.

Another possible bias of the measuring method is the dished contour of the polyethylene leading to an oblique introduction angle (10–15°) of the spatulas and overestimation of the lift-off of 3–4 %. It is our opinion that ligament-balancing surgery is not so fine-tuned that measurement-errors of this magnitude are clinically relevant.

It is widely accepted that good ligament balance is a cornerstone for good function and survival after TKA. However, it is problematic that there is no consensus on how stability should be measured intra-operatively. Many principles for evaluation of ligament balance during TKA have been developed, but they do not address the same problem. Different spacers, including trial components and blocks may assist in stretching the ligaments. The medial and lateral lift-off can then be measured by eye or indirectly by a computer in millimeters or degrees. Tensors and spreaders apply tension to the ligaments in a more or less controlled manner and electric instruments measures compressive loads. Most of these devices are expensive, add to the complexity of the surgery and are time consuming. Up to now, computer-assisted surgery has been the only established way to measure condylar lift-off intra-operatively. Although available for more than a decade, only a small part of TKAs are performed with computer assistance, probably due to high costs and prolonged operation time.

### Validation

The intraclass correlation coefficient was found to be high (0.88), indicating that the interobserver reliability is very good. This conclusion is strengthened by the high number of tests (96) and by the fact that the measurements were undertaken by five different assessors whose experience in total knee surgery ranged from 14 years to some months.

Ideally, validation of the new method should have been performed against an established gold standard. However, we believe that there is no absolute gold standard. Spreading devices, tensioners and spacer blocks allow measurements of gaps between osteotomies in a very different and non physiologic biomechanical situation without the prosthesis in place. Using a tensor Muratsu and Matsumoto found a decrease of as much as 5.3 mm in joint gap in extension and a reduction of varus ligament imbalance of 3.1° with the femoral trial prosthesis in place compared to measurements without [34]. We planed to compare our laxity measurements with those from computer-assisted surgery, but early trials found that this method overestimates the lift-off substantially. The reasons for this are unclear but might be related to the visco–elastic properties of bone.

### Ligament balancing

This part of the study was a tryout of the new method to measure ligament balancing on 100 TKAs. We found no statistically significant difference between ligament-balanced knees and non ligament-balanced knees in medial and lateral condylar lift-off in extension and 90° of flexion for varus and valgus knees (Table 4). No power analysis was performed and the number of knees tested is limited, so this conclusion must be drawn with caution.

Accepting 2 mm difference in medial and lateral condylar lift-off as a reasonable definition of medial–lateral symmetry, we found a high proportion of well-balanced knees, especially in extension (Fig. 2). It is, however, difficult to evaluate these results, because the limits for acceptable symmetry and laxity are so poorly defined in the literature. Further research is needed to find out if there is a connection between ligament-balance and function and prosthetic survival after TKA.

There is no consensus in the literature on how tight a TKA should be balanced. Our method for assessing ligament balance rests on the belief that some degree of visible lift-off is beneficial. This is in accordance with the findings of Edwards et al. [35]. They reported on 63 TKAs and found that lax knees showed better results in Hospital for Special Surgery Score (HSS) and pain, than stable knees. The stability was measured clinically at follow-up, 12–84 months after the operation. Kuster et al. [36] evaluated 22 patients with bilateral knee arthroplasties clinically and radiologically at a mean follow-up of 4.5 years. A modified HSS score (excluding laxity), varus and valgus stress X-rays in 30° of knee flexion, and the subjective outcome of both knees were compared. A knee was considered tight when it opened <4° and lax if it opened 4° or more on stress X-ray. Their results showed that patients with a preferred side felt significantly more comfortable on the laxer side.

Most orthopaedic surgeons agree that one goal for ligament balancing is to obtain rectangular gaps (that is equal medial and lateral lift-off). This goal was by far obtained in extension, but in flexion, it was some outliers (Fig. 2). Our tendency to obtain bigger gaps laterally may be due to the fact that we did not want to over-correct the varus knees and to the fact that native knees are looser laterally than medially in flexion. Tokuhara et al. [32] studied the flexion gap in 20 normal knees with MRI imaging. Under valgus stress, the mean medial gap was 2.1 ± 1.1 mm (0.2–4.2). When a varus stress was applied, the mean lateral gap was 6.7 ± 1.9 mm (2.1–9.2), indicating that the flexion gap is not rectangular but trapezoidal. The effect of such lateral laxity on prosthetic knee joints is unknown.

Another goal was to achieve equal extension and flexion gaps. As shown in Fig. 3, we were not able to reach this goal in the majority of the ligament-balanced knees. Nevertheless, the results were virtually the same for the not ligament-balanced knees, and there is some support in the literature that the flexion gap is bigger than the extension gap in normal, native knees. Van Damme et al. [27] quantified the ligament laxity in non-arthritic cadaver knees with a fluoroscopy assisted navigation system. In extension, the medial joint-line opening was on average 2.6 ± 1.0 mm and the lateral joint-line opening averaged 3.1 ± 0.8 mm. In 90° of flexion the medial join-line opening was on average 7.1 ± 1.4 mm and the lateral joint-line opening averaged 8.1 ± 1.0 mm.

When a mismatch between extension and flexion gap was present in our study, 98 % of the knees were tightest in extension. This is in contrast to the work of Griffin et al. [37] who found that less than 50 % of the knees were tightest in extension. We believe the reason why we generally obtained bigger flexion gaps is that we used a measured resection technique with anterior referencing and our policy to go down in size when forced to choose between femoral component sizes.

Recently Heesterbeek et al. [38] reported on varus–valgus laxity in extension and 70° of flexion in 49 TKAs implanted with a balanced gap technique. Ligament balancing was performed by releasing the tightest ligament first and laxity was measured with computer navigation while the knees were stressed to 15 Nm with a spring load. In extension, they found 2.6° (±1.1) (SD) valgus laxity and 2.8° (±1.6) varus laxity, and in flexion, 2.3 (±1.5)° valgus laxity and 2.7° (±1.8) varus laxity. Using a balanced gap technique, these authors succeeded in creating almost equal extension and flexion gaps, but their data are mean values and do not give any information on medial–lateral symmetry. Laxity outliers were not described and the results represent a selected group of patients with median age 60 years and knees with fixed varus- or valgus-alignment more than 10° and patients with BMI > 30 were excluded.

### Effect of patella eversion

In this study, all measurements of condylar lift-off were performed with the patella everted. There is some evidence in the literature show that patellar eversion affects ligament balance. Kamei et al. [39] assessed soft tissue balance by the gap technique in TKA, and found that gap inclination at 90° of flexion was higher with the patella in situ compared to with patella everted. Matsumoto and Muratsu measured the effect of ligament balance with a tensor and a navigation system. Their results are diverging with different results for cruciate retaining and posterior-stabilized knees [40]. Our method can easily be performed with the patella repositioned. An ongoing study is focusing on the effect of patellar eversion on condylar lift-off.

The present study has some limitations. First, our measuring tool do not distinguish between differences <1 mm, but ligament balancing surgery is not so exact that we feel a need for a more fine-tuned measuring device. Second, our method for measuring medial and lateral lift-off in extension is based on manual loading of the ligaments in valgus and varus. This is accounted for earlier in this paper. Third, the number of knees is limited, especially for valgus knees, thus firm conclusions cannot be drawn in the comparison between ligament-balanced and not ligament-balanced knees.

The strong points of this study are that it is prospective, the patients were recruited consecutively and inclusion criteria were well defined. The new method was tested on five different surgeons with different background and experience in total knee surgery. No data are missing.

In this study, the patients were operated with the measured resection technique, and therefore, less than perfect ligament balance becomes visible as lack of medial–lateral symmetry in condylar lift-off. Proper ligament balance is also important when the balanced gap technique is used, because in such cases, poor ligament balance in flexion can influence on the femoral component rotation [5, 6].

We conclude that our measuring device is reliable, simple, and easy to use. It enables the surgeon to document data on ligament balance objectively. Such data may be useful in further research on the relationship between ligament balance, function and survival of TKA.
